# Bidirect effects from cisplatin combine with rosmarinic acid (RA) or hot water extracts of *Glechoma hederacea* (HWG) on renal cancer cells

**DOI:** 10.1186/s13020-020-00358-2

**Published:** 2020-07-29

**Authors:** Su-Tze Chou, Bing-Ying Ho, Yu-Ting Tai, Chun-Jen Huang, Wen-Wan Chao

**Affiliations:** 1 Department of Food and Nutrition, Providence University, Taichung, 433 Taiwan; 2 Department of Anesthesiology, Wan Fang Hospital, Taipei Medical University, Taipei, 116 Taiwan; 3 Department of General Surgery, Wan Fang Hospital, Taipei Medical University, Taipei, 116 Taiwan; 4 Department of Nutrition and Health Sciences, Kainan University, No.1 Kainan Road, Luzhu Dist, Taoyuan City, 33857 Taiwan, ROC

**Keywords:** *Glechoma hederacea*, Rosmarinic acid, Cisplatin, 786-O cells, FAK

## Abstract

**Background:**

Cisplatin (CDDP) is a chemotherapeutic drug which also causes adverse side effects. *Glechoma hederacea* is a traditional Chinese herb belonging to the Labiatae family and has many biological activities. Our previous study indicated that rosmarinic acid (RA) was the most abundant phytochemical in *G. hederacea*. However, the antioxidant or anti-inflammatory effects of the combined treatment of *G. hederacea*, RA and CDDP on human renal cell carcinoma (RCC) 786-O cells have not been clearly demonstrated. We aimed to investigate the bioefficacy of hot water extracts of *G. hederacea* (HWG) and RA in inhibiting RCC 786-O cell activity and its synergism with CDDP against metastatic renal cancer cell.

**Methods:**

Bioactivities of the combination treatment of HWG, RA, HWG/CDDP and RA/CDDP were assessed using the MTT assay and transwell migration, and the crude extract/compound efficacy was evaluated using wound healing migration assays, flow cytometry and western blotting.

**Results:**

Our study indicates that CDDP inhibits 786-O cell proliferation and migration and HWG and RA protect against these effects. On the other hand, HWG and RA demonstrate a low cytotoxic effect in human renal proximal tubular epithelial cell line -2 (HK-2 cells). Cell cycle analysis found that HWG/CDDP and RA/CDDP combined treatment exerted cytotoxicity by inducing G2/M arrest and apoptosis. RA in combined with CDDP significantly inhibiting the expression of p-FAK (Tyr 925) in RCC 786-O cells in vitro.

**Conclusion:**

We propose that the inhibition of RA on RCC 786-O cell invasion and migration may partly occur through the downregulation of FAK phosphorylation. The HWG/CDDP and RA/CDDP combined treatments may be effective strategies for intervention of RCC 786-O cell activity.
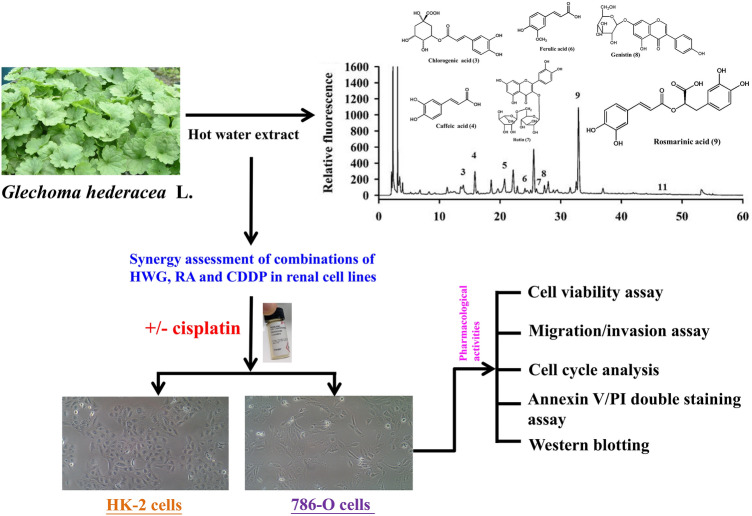

## Background

Renal cell carcinoma (RCC) is the most common type of kidney malignancy and accounts for ~ 3% of tumors in adults. However, only 2% RCC cases are associated with inherited gene mutations. It is also associated with a high metastatic potential [1, 2]. RCC comprises several histological cell types, including clear cell RCC, papillary RCC, chromophobe RCC, and collecting duct RCC. Currently five traditional and well-defined subtypes of RCC are known: conventional clear cell RCC, papillary (types 1 and 2) RCC, chromophobe RCC, carcinoma of the collecting ducts of Bellini, and unclassified RCC and these subtypes represent the majority of RCC cases diagnosed. Clear cell RCC (ccRCC) is the most common subtype of renal cancer and accounts for approximately 70–75% of cases, so it cannot be assumed that all RCC derived cell lines represent ccRCC [3–5].

Cisplatin (*cis*-diamminedichloroplatinum, CDDP) is one of the most potent platinum-based anticancer agents [6]. Its mode of action is mediated by its interaction with DNA to form DNA adducts, which activate several signal transduction pathways leading to cell death [7, 8]. However, the development of chemotherapeutic resistance to CDDP results in treatment failure. Phytochemicals are naturally occurring plant-based compounds that can augment the anti-cancer activity of CDDP, with minimal side effects. Combination therapy, which is a treatment modality combining two or more therapeutic agents is a cornerstone in cancer therapy and has provided highly effective results [9–12].

*Glechoma hederacea* is a traditional Chinese herbal medicine belonging to the Labiatae family. We have reported that hot water extracts of *G. hederacea* (HWG) possess antioxidant activity owing to the presence of polyphenolic compounds [13, 14]. Our study indicated that rosmarinic acid, chlorogenic acid, caffeic acid, rutin, genistin, and ferulic acid were the most abundant phytochemicals in HWG and possess potent antioxidant and anti-inflammatory properties [15]. Supporting evidence indicated that *G. hederacea* extracts possess various biological activities including depigmentation, anti-melanogenic, anti-tumor, antioxidative, hepatoprotective, and anti-inflammatory activity [16–20].

Rosmarinic acid (RA), the ester of 3, 4-dihydroxyphenyllactic and caffeic acids is considered to be one of the most important polyphenols. Studies have further ascertained the anti-microbial, immunomodulatory, anti-diabetic, anti-allergic, anti-inflammatory, hepato- and renal-protectant effects of RA, as well as its beneficial effects on skin afflictions [21–23].

New anticancer approaches can be explored using Traditional Chinese medicinal (TCM) plants which are an excellent source of chemotherapeutic agents with various biological activities and great potential therapeutic value. Natural products have always played a pivotal role in anticancer drug discovery with majority of the anticancer drugs being either pure natural products or their synthetic/semisynthetic derivatives [24, 25]. Medicinal plants and natural herbal products are often administered in combination with chemotherapeutic drugs and conventional therapy to increase their potential antioxidant activity and provide better protection against their nephrotoxic effect [26, 27].

The result of the phytochemical effect of HWG and RA in combined with CDDP on RCC 786-O cells has not been clearly demonstrated. This study investigated the bioefficacy of HWG and RA in inhibiting RCC 786-O cell activity and its synergism with CDDP against metastatic renal cancer. The effect of this combined treatment on cell growth, cell proliferation, cell cycle, cell death mechanism and cell migration was monitored. In addition, cell cycle- and apoptosis-regulating proteins were assessed by Western blotting.

## Materials and methods

### Preparation of *G. hederacea* extracts

Naturally grown *G. hederacea* was obtained from Taichung City, Taiwan. A voucher specimen was identified by Dr. Bing-Shiunn Chen and deposited in the Department of Horticulture, National Chung Hsing University, Taichung City, Taiwan (No. NCHU-2016-001). The *G. hederacea* extracts were prepared in accordance with our previously reported procedures [14, 15]. Briefly, the whole plants of *G. hederacea* were cut into small pieces and extracted at 1:50 (w/v) dilutions in distilled water (100 °C for 3 h; HWG). The decoctions were filtered, lyophilized, and stored at − 70 °C until use.

### Chemicals and reagents

DMEM-F12, RPMI medium 1640 (Gibco/BRL), fetal bovine serum (FBS), penicillin, streptomycin, trypsin–EDTA were obtained from Invitrogen. Rosmarinic acid, cisplatin (CDDP), 3-(4,5-dimethylthiazol-2-yl)-2,5-diphenyltetrazolium bromide (MTT), and dimethyl sulfoxide (DMSO), isopropanol were from Sigma Chemical (St. Louis, MO). Primary antibodies against PARP (#9542), β-actin (#4970), and phospho-FAK/Y925 (#3284) were purchased from Cell Signaling Technology, Inc.

### Cell cultures

Human renal proximal tubular epithelial cell line -2 (HK-2 cells) was kindly provided by Professor Yi-Hsien Hsieh (Institute of Biochemistry, Microbiology and Immunology, Chung Shan Medical University, Taichung, Taiwan). Human renal cell carcinoma (RCC 786-O) cells were purchased from Bioresource Collection and Research Center (Hsinchu, Taiwan).

HK-2 cells were maintained in DMEM-F12 containing 10% FBS, 100 units/mL penicillin and 100 mg/mL streptomycin solution at 37 °C and 5% CO_2_. RCC 786-O cells were maintained in RPMI-1640 containing 10% FBS, 100 units/ml penicillin and 100 mg/mL streptomycin in a humidified atmosphere with 5% CO_2_ at 37 °C.

### 3-(4,5-Dimethylthiazol-2-yl)-2,5-diphenyltetrazolium bromide (MTT) assay

The effect of CDDP combined with HWG or RA on the viability of HK-2 cells and RCC 786-O cells were determined using the MTT assay. Cells were seeded in growth medium in 96-well plates for 24 h, treated with different concentrations of CDDP combined with HWG or RA for 48 h, following the addition of MTT (Invitrogen; M6494) reagent for 4 h. The formazan precipitate was dissolved in DMSO, and absorbance was measured at 570 nm using a microplate reader ELx800 (BioTek Instruments, Inc., Winooski, VT, USA).

### Transwell migration assay

The in vitro cell migration assay was optimized according to Ho et al. [28]. The cell migration assays were performed in a Millicell cell culture insert system (Millipore; PIBP01250). The 786-O cells (2 × 10^4^ in 100 μL of growth medium without FBS) were placed in the upper chamber and 400 μL of growth medium with 10% FBS was placed in the lower chamber. The cells were treated with agents as described in the figures and incubated at 37 °C for 16 h. Following the incubation, the insert membranes were fixed with 100% ethanol for 30 min. The cells on the upper surface were removed with cotton-tipped swabs, and the migrated cells on the lower surface were stained with 4′,6-diamidino-2-phenylindole (DAPI, Sigma; D9452). The cell nuclei were stained with DAPI and appeared as blue dots in the a fluorescent microscopic image.

### Wound healing migration assay

The in vitro wound healing migration assay was optimized according to Ho et al. [28]. Wound healing and migration assays were done by seeding the 786-O cells (3.5 × 10^4^/well) into the Ibidi-silicone insert (Ibidi; Cat. #81176, Germany) and incubated for attachment. This insert allows the formation of a well‐defined edge without physically scratching or wounding the cell monolayer after removal of the inserts. In order to monitor cell movement within the wounded area following their incubation with diverse concentrations of cisplatin combined with HWG or RA, three wounds were photographed immediately after picking up the insert (T_0_) and after 6 h. The endpoint of the assay was measured by calculating the reduction in the width of the wound after 6 h and comparing it to T_0_ with the non-treatment control group set at 100%.

### Cytofluorimetric analysis

Cell cycle analysis was performed as described by Ho et al. [28]. Adherent treated and control 786-O cells were harvested 48 h after the treatment as described in the figures. Cells were washed with PBS, fixed in 70% cold ethanol, resuspended in a buffer containing 0.05 mg/mL propidium iodide (PI, Sigma, Cat. #81845) for cell cycle analysis using a Cytoflex flow cytometer (Beckman Coulter, Inc., Indianapolis, IN, USA). A minimum of 10,000 events were collected and analyzed. The treated cells were analyzed by FITC annexin V apoptosis detection kit (BioLegend, Cat. #640914) to examine the induction of apoptosis and necrosis.

### Western blotting

Total cellular proteins were prepared from test cells according to Chiang et al. [29]. Protein concentration was determined by the Bradford method (Bio-Rad Laboratories, Hercules, CA). After being seeded in 7-cm dishes at a density of 1 × 10^6^ cells/dish for 24 h, 786-O cells were then treated with agents as described in the figures. On the day of harvest, the whole-cell lysates were extracted with 1 × radioimmunoprecipitation lysis buffer (Millipore, Cat. #20-188) containing 1 × protease inhibitor cocktail (Milipore, Cat. #20-201). The protein extracts were resolved by sodium dodecyl sulfate–polyacrylamide gel electrophoresis and subsequently transferred to polyvinylidene difluoride membrane (Pall, Cat. #66543) by electroblotting. The membranes were blocked with 5% bovine serum albumin in Tris-buffered saline (TBST) buffer (Tris-buffered saline with Tween 20, 25 mM Tris–HCl, 125 mM NaCl, 0.1% Tween 20) for 1 h at room temperature and incubated overnight with primary antibody at 4 °C and then with horseradish peroxidase-conjugated secondary antibody for 1 h at room temperature. Intensive wash with TBST buffer was performed after each incubation. The immune complexes were visualized using enhanced Chemiluminescence (ECL) Reagent Plus (Milipore, Cat. # MI-WBKLS0500) on the Syngene G: Box chemi XL gel documentation system (Syngene, Cambridge, UK) according to the manufacturer’s instructions. Quantification of Western blot band intensities was performed using ImageJ software (ImageJ bundled with 64-bit Java 1.8.0_112, National Institutes of Health, Bethesda, MD, USA) downloaded from https://imagej.nih.gov/ij/download.html.

### Statistical analysis

All assays were conducted at least three times with three different sample preparations. All data were expressed as the mean ± standard deviation (SD). Analysis of variance was performed using the GraphPad Prism 7. One-way ANOVA and Scheffe’s method were used to analyze the differences between the mean values. Differences with *P* < 0.05 were considered statistically significant.

## Results

### Inhibitory effects of HWG, RA and CDDP on the growth of HK-2 and 786-O cells

**Fig. 1:**
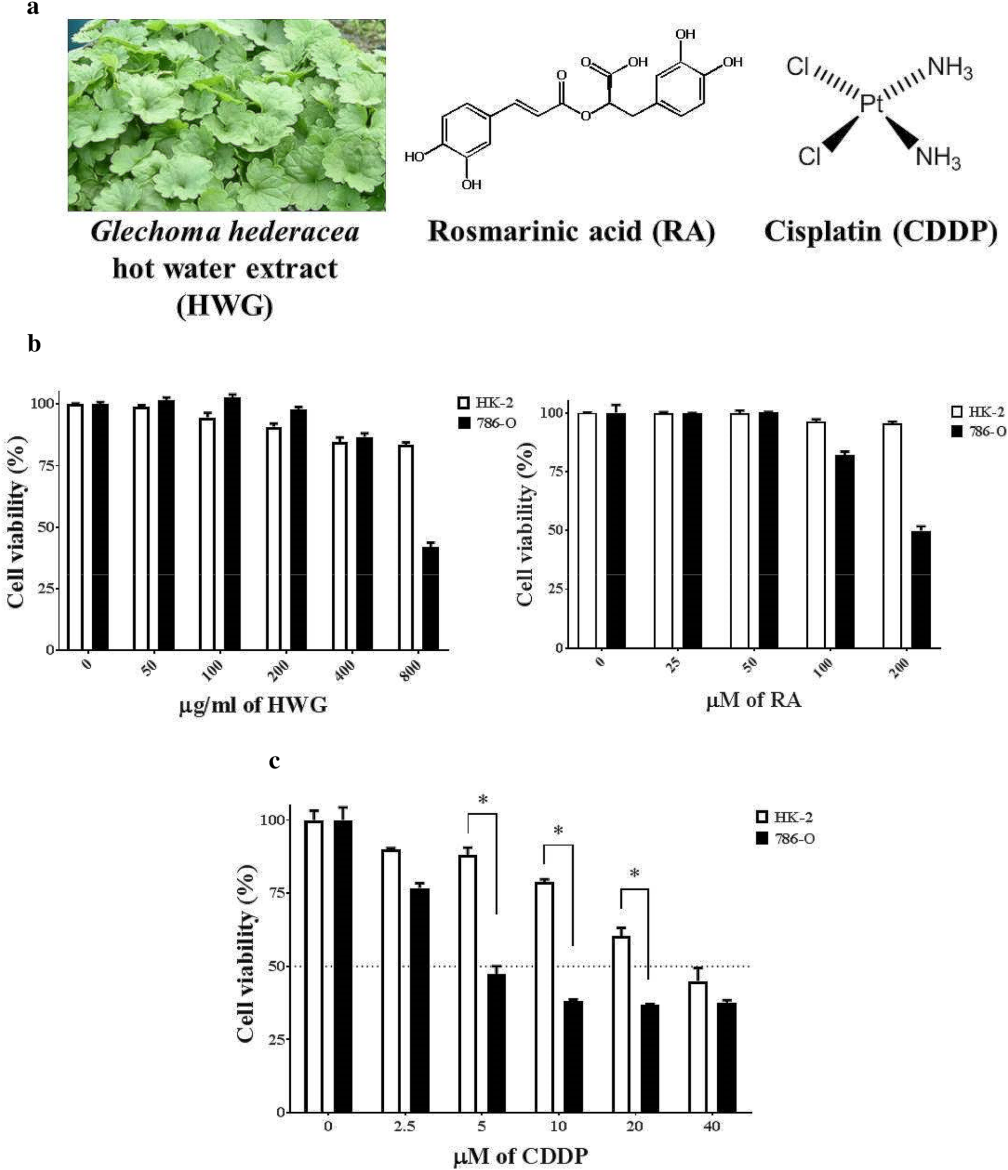
Effect of hot water extract of *Glechoma hederacea* (HWG), rosmarinic acid (RA) on HK-2 and 786-O cells viability after treatment with cisplatin (CDDP). **a** Chemical structures of RA and CDDP. HK-2 and 786-O cells were exposed to various concentrations of HWG (50–800 μg/mL), RA (25–200 μM) (**b**) and CDDP (2.5–40 μM) (**c**) and cell viabilities were measured 48 h later using the MTT assay. The data represent the mean ± SD from three independent experiments. ^∗^*P *< 0.05

To study the effects of HWG, RA and CDDP on the proliferation of HK-2 cells, 786-O cells were exposed to different concentrations of HWG, RA and CDDP for 48 h and monitored using the MTT assay. Figure 1 shows the structures of compounds RA and CDDP. Figure 1 shows that HWG, RA and CDDP dose-dependently inhibit 786-O cell proliferation. HWG concentrations of 0, 50, 100, and 200 μg/mL, RA concentrations of 0, 25, 50, and 100 μM and CDDP concentrations of 5 μM were used for further in vitro experiments. Meanwhile, CDDP showed toxicity on human RCC 786-O cells. Our study demonstrated that CDDP dose-dependently inhibits 786-O cell and HK-2 cells proliferation.

### HWG, RA sensitizing CDDP activity inhibiting migration of 786-O cells

**Fig. 2:**
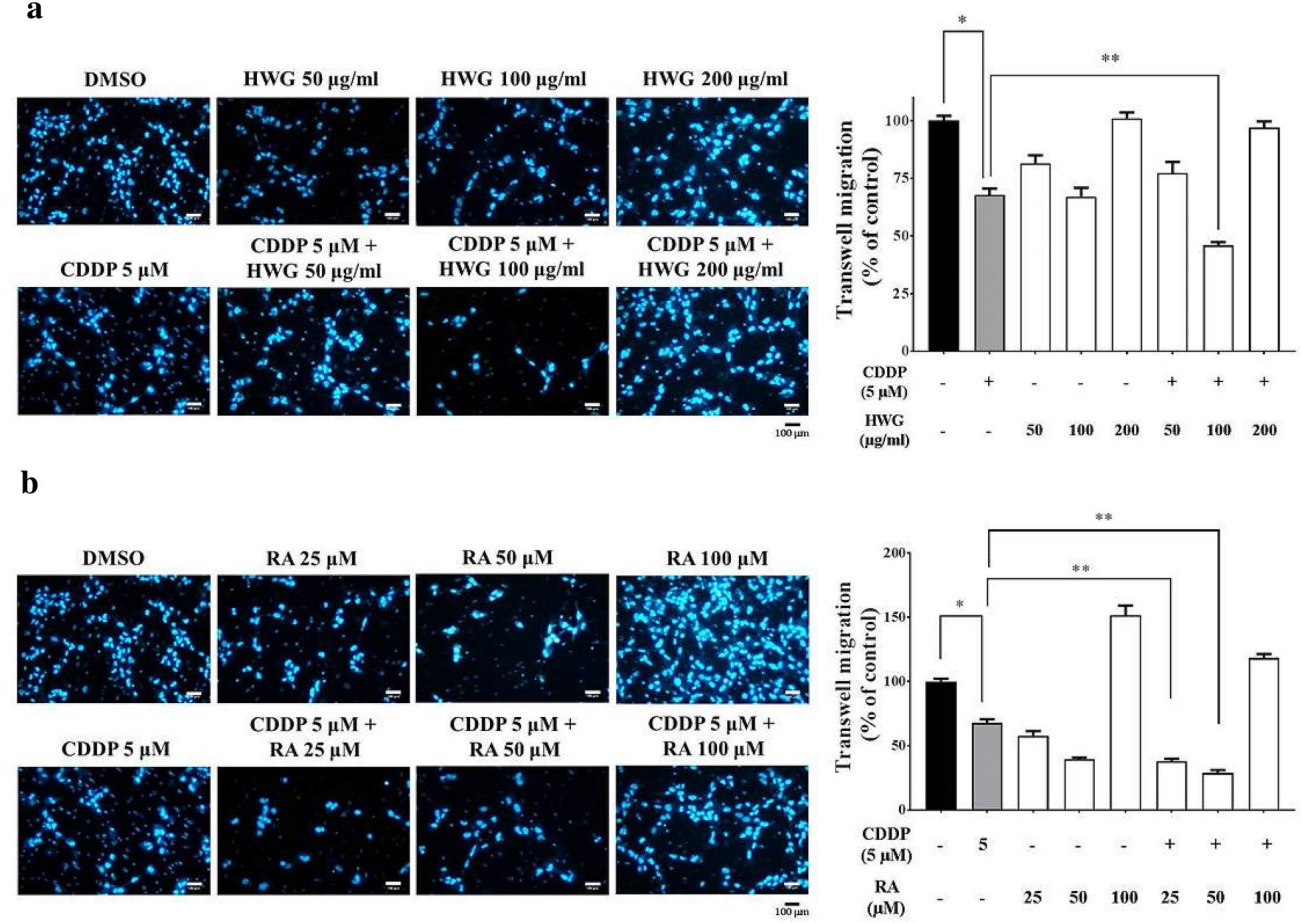
HWG and RA sensitizing CDDP activity inhibiting migration of RCC 786-O cells. RCC cells were incubated with various concentrations (50–200 μg/mL) of HWG alone or in combined with CDDP (5 μM) (**a**); (25 ~ 100 μM) of RA alone or in combined with CDDP (**b**). The migration abilities were determined using a Matrigel invasion assay. The cells in the lower surface of the Borden chamber were stained and photographed under a light microscope at 400× magnification. The quantification of migration and invasion abilities are shown in a histogram. Data are presented as the mean ± SD of at least three independent experiments. ^∗^*P* < 0.05, ^∗∗^*P* < 0.01 compared with the CDDP

A crucial characteristic of metastasis is the migration and invasion of tumor cells. Treating RCC 786-O cells with various concentrations of HWG for 16 h showed that HWG did not inhibit the invasion of RCC cells in a dose-dependent manner. On the other hand, HWG in combined with CDDP inhibited the invasion of RCC cells, especially at a concentration of more than 100 μg/mL (Fig. 2a). Treating RCC 786-O cells with various concentrations of RA for 16 h showed that RA in combined with CDDP inhibited the invasion of RCC cells, especially at concentrations of more than 25 and 50 μM (Fig. 2b).

### HWG, RA inhibiting wound healing migration of 786-O cells

**Fig. 3:**
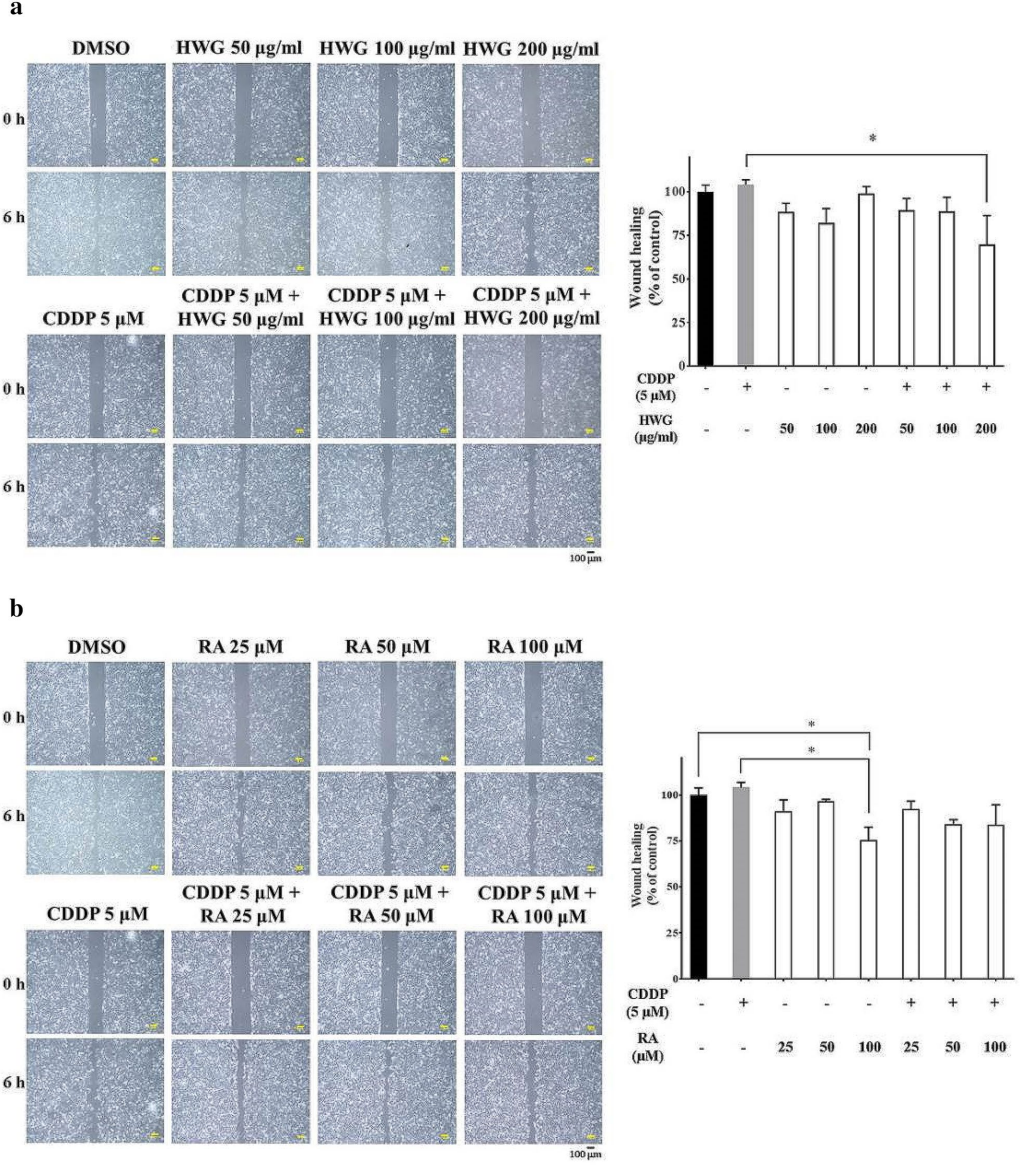
Effect of HWG and RA on the decreased RCC 786-O cell migration capacity caused by CDDP treatment. Scratch wound healing assay was used to explore cell migration capacity. **a** Monolayers of RCC cells treated with HWG (50–200 μg/mL) alone or in combined with CDDP (5 μM) were scraped and the number of cells in the denuded zone was photographed and quantified after indicated times (0, 6 h). **b** Monolayers of RCC cells treated with RA (25–100 μM) were scraped and the number of cells in the denuded zone was photographed and quantified after indicated times (0, 6 h). Quantitative assessment of the mean number of cells in the denuded zone represents the average of three independent experiments ± S.D. ^∗^*P* < 0.05 compared with CDDP

Treating RCC 786-O cells with various concentrations of HWG for 6 h showed that HWG did not inhibit the wound-healing migration of RCC cells in a dose-dependent manner. HWG in combined with CDDP inhibiting the wound healing migration of RCC cells, especially at a concentration of more than 200 μg/mL (Fig. 3a). Treating RCC 786-O cells with various concentrations of RA for 6 h showed that RA (100 μM) inhibited the wound healing migration of these RCC cells. But, RA in combined with CDDP did not inhibiting the wound healing migration of RCC cells (Fig. 3b).

### The synergistic effect of HWG, RA combined with CDDP induced G2/M cell cycle arrest in 786-O cells

**Fig. 4:**
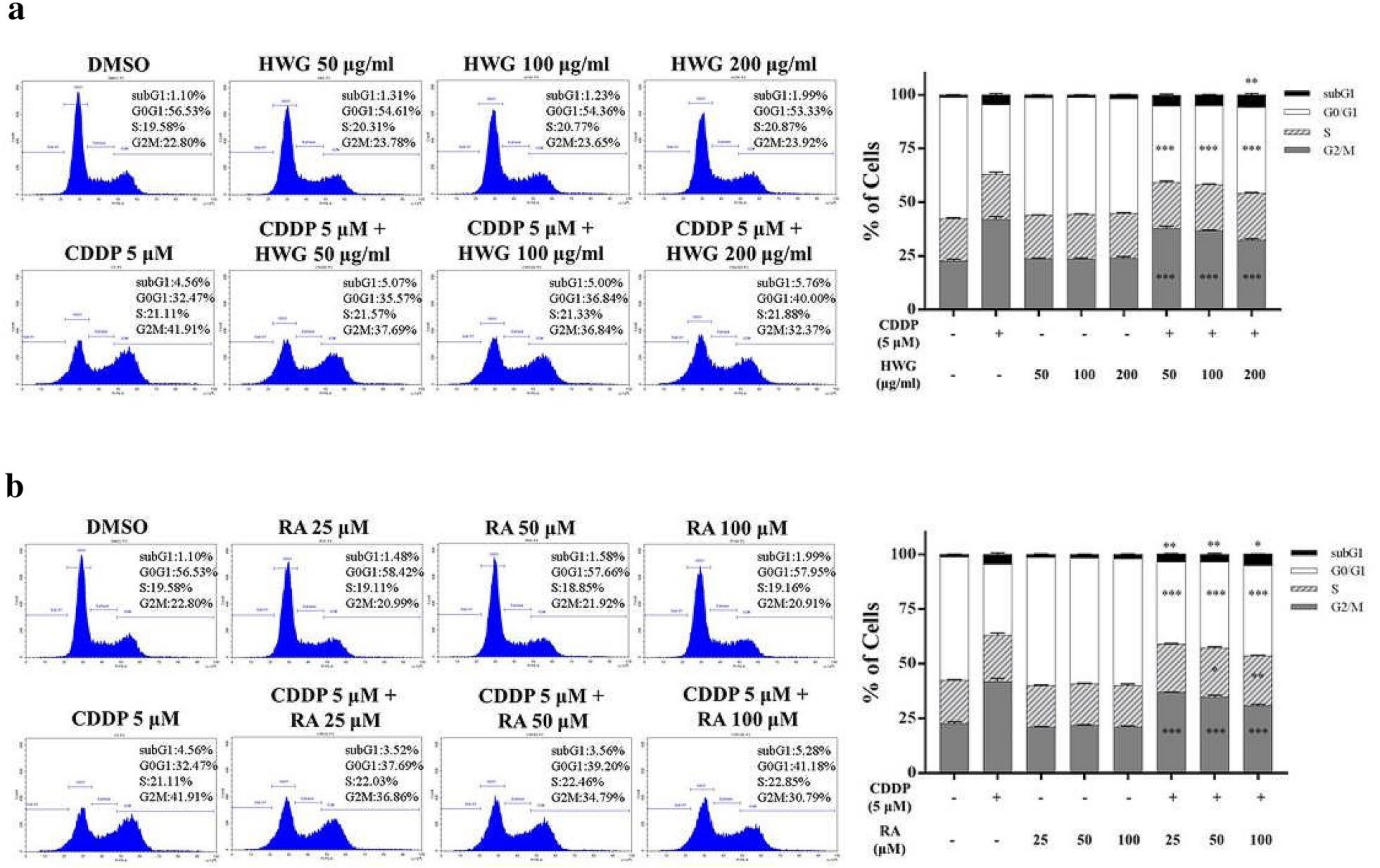
Effects of HWG, RA and CDDP synergistically arrest the cell cycle at G2/M phase and apoptosis in RCC 786-O cells. 786-O cells were treated with the vehicle control, CDDP (5 μM) alone, HWG (50 ~ 200 μg/mL), RA alone, or HWG combined with CDDP (**a**), and RA (25 ~ 100 μM) combined with CDDP (**b**) incubated for 48 h. Cell cycle distribution based on DNA content was analyzed through flow cytometry. Different cell phases were plotted as the percentage of total events. Data are presented as the mean ± SD of at least three independent experiments. ^∗^*P * < 0.05, ^∗∗^*P * < 0.01, and ^∗∗∗^*P * < 0.001 compared with CDDP

Cell cycle is an essential process for the growth, development and differentiation of mammalian cells. In particular, many plant constituents block cell cycle progression at different stages of the cell cycle (G2/M, S or G0/G1phases) and thereby induce apoptotic cell death. Cell cycle arrest is an important factor for many anticancer agents [30]. To explore the mechanism involved in HWG, RA-induced inhibition of RCC 786-O cell proliferation, the effects of HWG, RA on the cell cycle arrest were examined. 786-O cells were incubated with various concentrations of HWG or in combined with CDDP for 48 h. The G2/M phase arrest increased from 23.78% to 37.69%, 23.65% to 36.84% and 23.92% to 32.37% in 786-O cells in combined with CDDP compared to HWG only in a dose-dependent manner (Fig. 4a).

Furthermore, we assessed various concentrations of RA or in combined with CDDP for 48 h. The G2/M phase arrest increased from 20.99% to 36.86%, 21.92% to 34.79% and 20.91% to 30.79% in 786-O cells in combined with CDDP compared to RA only, in a dose-dependent manner (Fig. 4b). Thus, HWG and RA inhibit 786-O cell proliferation, as well as arrest cell cycle progression in the G2/M phase.

### Treatment with HWG, RA, and their combined with CDDP induces apoptosis in 786-O cells

**Fig. 5:**
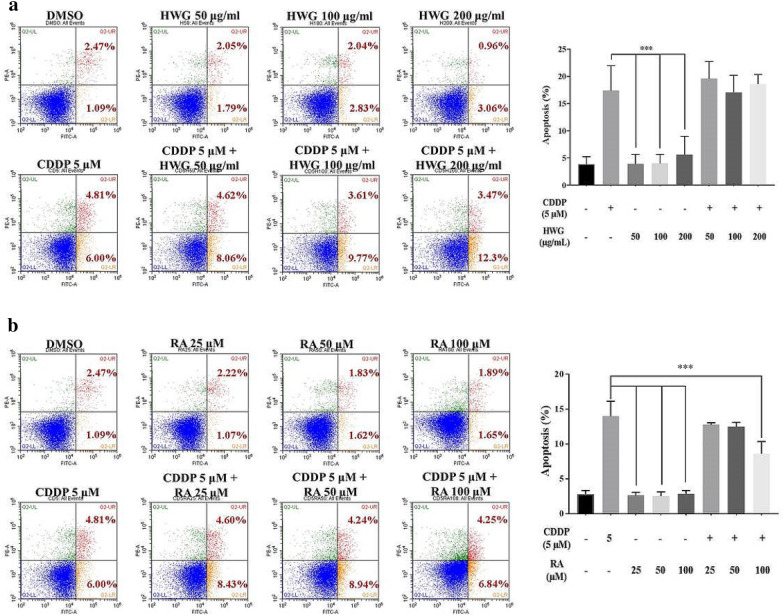
Effect of HWG and RA on CDDP-induced apoptosis in RCC 786-O cells, measured 48 h later using AnnexinV-FITC/PI assay. **a** The percentages of apoptotic cells after HWG (50–200 μg/mL), **b** RA (25–100 μM) treatment in the absence or presence of CDDP (5 μM) are indicated at the top of the figure (upper panel). All percentages are graphed (lower panel). The data represent the mean ± SD of those of three independent experiments. ^∗^*P* < 0.05 compared with the CDDP

**Fig. 6:**
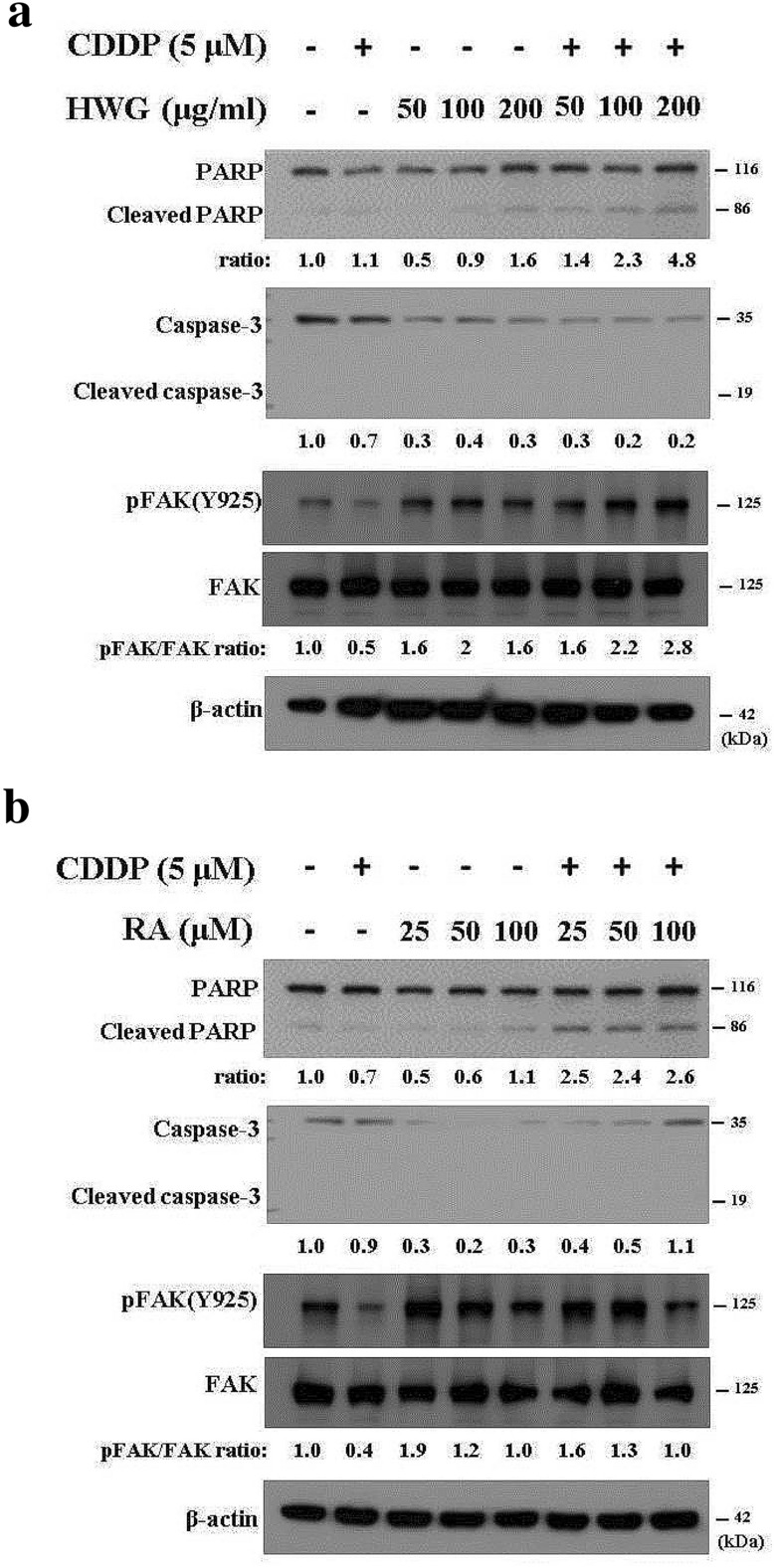
Effects of HWG and RA, and in combined with CDDP induced the expression levels of PARP and FAK protein in RCC 786-O cells. **a** Western blot analysis of PARP, cleaved PARP, FAK, p-FAK in 786-O cells after 48 h of treatment with HWG. **b** Western blot analysis of PARP, cleaved PARP, FAK and p-FAK in 786-O cells after 48 h of treatment with RA. Western blotting with PARP, cleaved PARP, FAK and p-FAK (Tyr925) antibodies, with anti-β-actin as an internal control. Similar results were obtained from three repeated and independent experiments

To assess the induction of apoptosis by HWG, RA, and their combined with CDDP, 786-O cells were treated with the extract/compound, and apoptosis was determined by flow cytometry. The percentages of early and later apoptotic cells were shown in the lower right (LR) and upper right (UR) quadrant of the histograms respectively. The total percentage of apoptotic cells (UR + LR) increased from 3.56% in non-CDDP treated 786-O cells to 10.81% (Fig. 6a, b). Treatment with HWG alone did not induce apoptosis (10.81% vs. 3.84%, 4.87%, 4.02%) after 48 h in 786-O cells. The percentage of apoptotic cells before and after HWG and combined with CDDP treatment was 10.81% vs. 15.77% at 48 h for 786-O cells (Fig. 5a).

On the other hand, 786-O cells treated with RA alone did not induce apoptosis (10.81% vs. 3.29%, 3.45%, 3.54%) after 48 h. The combined treatment of RA (100 μM) and CDDP induced significantly less apoptosis (10.81% vs. 13.03%, 13.18%, 11.09%) at 48 h (Fig. 5b).

### HWG and RA altered the expression level of phosphorylated focal adhesion kinase (FAK) in 786-O cells

We investigated the molecular regulation of cell migration by HWG, RA, and their combination with CDDP through Western blot. The protein levels of cleaved-PARP and PARP were detected by Western blot analysis. The data demonstrated that CDDP combination treatment induced high expression of cleaved-PARP protein compared with the control group, in a dose-dependent manner (Fig. 6a, b). Together, these results indicated that HWG, RA, and their combined with CDDP significantly induced apoptosis by PARP.

We then examined the variation in FAK levels in these cells by using antibodies directed against the FAK phosphorylation site Tyr925. As illustrated in Fig. 6, HWG in combined with CDDP increased FAK phosphorylation (Fig. 6a). The Western blot results are presented in Fig. 6b, which demonstrate that RA in combined with CDDP significantly inhibited the expression of p-FAK (Tyr 925) in RCC 786-O cells. On the basis of these results, we propose that the inhibition of RA in RCC 786-O cell invasion and migration may partly occur through the downregulation of FAK phosphorylation.

## Discussion

Cancer is one of the most common life-threatening diseases in the 21st century with rising incidence rates and progressively reducing age of the afflicted patients. There are evidences that inflammation and cancer development has strong interaction [1]. Renal cell carcinoma (RCC) is the most common type of kidney malignancy in adults that is established in the renal proximal convoluted tubules. Among them, the clear cell RCC (ccRCC) is the most common type, comprising approximately 70%–80% of the RCC cases. The selection of 786-O cells in this study was therefore based on the clinical and epidemiological data of RCC as well as their specific cellular properties. Thus, 786-O cells are considered to be an appropriate model for RCC and have been widely used in different types of RCC research such as proliferation, apoptosis, migration and invasion, metastasis or therapy resistance studies [31–33].

In addition to analyzing HWG, RA and CDDP toxicity in normal cells, we exposed HK-2 cells to the same concentrations as 786-O cancer cells. HK-2 cells were obtained from a primary proximal tubular cell culture of the normal adult human renal cortex and exposed to a recombinant retrovirus containing HPV 16 E6/E7 genes. These cells retain a phenotype indicative of well-differentiated primary proximal tubular epithelium and retain functional characteristics of proximal tubular epithelium [34]. Interestingly, HWG and RA displayed a very low efficiency in HK-2 cells, indicating that HWG and RA are less toxic for normal cells than for 786-O cancer cells. On the contrary, CDDP highly reduced the growth of 786-O cancer cells than HK-2 cells.

Our study shows that HWG, RA and CDDP dose-dependently inhibit HK-2 and 786-O cell proliferation. Meanwhile, CDDP showed toxicity on human RCC 786-O cells. Since reports of CDDP’s cytotoxic effect vary across studies and may depend on the cell types, our study used two cell types HK-2 and RCC 786-O cells. The concentration of CDDP that reduced cell viability by about 50% compared to RCC 786-O cells was less than that of HK-2 cells, indicating RCC 786-O cells are more sensitive to CDDP. Thongnuanjan et al. reported that CDDP is transported into renal proximal tubular cells via the renal organic cation transporter 2 (OCT2) but not so in HK-2 cells [35].

Chinese medicines have been widely used as health-promoting food ingredients and supplements for the treatment of cancer. The combination of anti-tumor agents should be able to produce synergistic or additive therapeutic efficacies, reduced side effects and minimal drug resistance. Experimental strategies that investigate combinational therapy of CDDP with natural plant extracts/plant-derived agents to maintain the desired antitumor efficacy have been reported [9, 36, 37]. In this study, HWG in combined with CDDP inhibits the invasion of these RCC cells, especially at a concentration of more than 100 μg/mL and inhibited the wound-healing migration of these RCC cells, especially at a concentration of more than 200 μg/mL. RA in combined with CDDP inhibited the invasion of these RCC cells, at a concentration of more than 25 and 50 μM. But, RA in combined with CDDP did not inhibit the wound healing migration of these RCC cells. Here, we demonstrate the preventive and therapeutic effect of HWG and RA alone as well as their synergism with CDDP, against RCC 786-O metastasis. Rosmarinic acid’s anti-cancer activity was not restricted to the colon, but also included skin cancer and melanoma, pancreatic cancer, breast cancer, lung cancer, leukemia, hepatoma and ovarian cancer [21]. Fisetin (3,3′,4′,7-tetrahydroxyfavone), a naturally occurring flavonoid commonly found in plants is effective against cancer, and its possible mechanisms includes suppression of proliferation and metastasis of RCC through upregulation of MEK/ERK-Targeting CTSS and ADAM9 [32].

Our in vitro mechanistic study shows that HWG/CDDP and RA/CDDP synergistically inhibit or disrupt RCC 786-O cell motility and cell-cycle machinery. Thus, HWG and RA inhibit 786-O cell proliferation, as well as arrest cell cycle in the G2/M phase. Induction of apoptosis by HWG and RA, and in combined with CDDP was assessed in 786-O cells through treatment with the extract/compound followed by flow cytometric analysis. We conducted an Annexin V-FITC/PI double staining assay to investigate whether HWG/CDDP and RA/CDDP combination reduced RCC 786-O cells viability via apoptosis induction. Only RA in combined with CDDP induced significantly less apoptosis at 48 h. Several reports have previously described that CDDP induces S- and G2/M-phase arrest in a sequential manner [38]. Apoptosis is an essential cell process in the homeostasis of multicellular organisms, and its dysregulation has been involved in many human tumors [39, 40]. G2/M phase cell cycle arrest is one of the most prominent checkpoints of many anticancer agents which can reduce proliferation and then induce apoptosis by inhibiting the segregation of damaged chromosomes during mitosis [41–43].

CDDP induces apoptotic cell death as evidenced by increased cleavage of caspase-3 and PARP. During apoptosis, cleavage of PARP by caspase-3 in response to DNA strand breaks has become a hallmark of this type of cell death. Our data indicates that HWG and RA and their combined with CDDP significantly induces apoptosis by PARP. Thus, the protein levels of cleaved-PARP and PARP were assessed by Western blot analysis. The data demonstrates that cisplatin induced high expressed of cleaved-PARP compared with the control group. PARP-1 is a poly-(ADP-ribosylated) enzyme necessary for DNA repair. Thus, cleaved PARP-1 is considered to be a remarkable marker of apoptosis. PARPs modify target proteins post-translationally with poly (ADP-ribose) (PAR) or mono (ADP-ribose) (MAR) using NAD^+^ as substrate, and play a central role in renal epithelial cell apoptosis during cisplatin nephrotoxicity [44, 45].

Focal adhesion kinase (FAK) is a non-receptor tyrosine kinase that plays a vital role in focal adhesion. It is regulated by multiple phosphorylation sites, tyrosine 397, Tyr 576/577, and Tyr 925. Wound healing experiments were done to evaluate how Tyr-925 phosphorylation affects cell migration. FAK activation, results in FAK phosphorylation at Tyr-397 (Tyr(P)-397), promotes Src family protein-tyrosine kinase binding to the FAK Tyr(P)-397 site, and facilitates the formation of the FAK-Src signaling complex that results in the secondary phosphorylation of FAK at Tyr-861 and Tyr-925. FAK, a protein tyrosine kinase, is overexpressed in several cancers and promotes cancer progression and metastasis [46, 47]. Our Western blot result demonstrated that RA in combined with CDDP significantly inhibited the expressions of p-FAK (Tyr 925) in RCC 786-O cell, not observed in HWG.

Cell migration is implicated in various processes including embryo-genesis, tissue regeneration, wound healing, and tumor progression. During this process, cells interact with the microenvironment in part through focal adhesions. FAK clusters at focal adhesion structures regulates cancer-associated processes, including adhesion, migration and invasion [48, 49]. Moreover, cell cycle analysis revealed that FAK inhibition induced G1 arrest in 786-O cells. On the basis of these results, we propose that the inhibition of RA on RCC 786-O cell invasion and migration may partly occur through the downregulation of FAK phosphorylation.

RA is a highly valued natural phenolic compound that is very commonly found in plants families *Lamiaceae* and *Boraginaceae*. In a review of the biological effects of RA, Moore et al. concluded that it could be used as a phytochemical to induce apoptosis. RA can reduce survival of cancer cell lines such as HT-28, MCF-7, DU145 or MKN45, among others. The administration of RA in colon cancer cell lines (HT-29) reduced a transcription factor through inhibition of activator protein-1 (AP-1), which is responsible for the activation of COX-2. The anti-cancer activity of RA was not restricted only to the colon, but also included skin cancer and melanoma, pancreatic cancer, breast cancer, lung cancer, leukemia, hepatoma and ovarian cancer [21, 50, 51].

Organic compounds that have been discovered in natural sources, such as plants, animals, and microorganisms, have been an inspiration for drug development. Newman and Cragg concluded that more than 50% of all modern clinical drugs have their origin in natural products [52]. Many phytochemicals possess various anticancer activities with minor side effects in comparison with traditional anticancer drugs. Phytochemicals also promote the efficacy of chemotherapy and/or radiotherapy. Phytochemicals exert antitumor effects via distinct mechanisms. They selectively kill rapidly dividing cells, target abnormally expressed molecular factors, remove oxidative stress, modulate cell growth factors, inhibit angiogenesis of cancerous tissue, and induce apoptosis. For example, some polyphenols (resveratrol, gallocatechins), flavonoids (methoxy licoflavanone, alpinumisoflavone), and brassinosteroids (homocatasterone and epibrassinolide) exert anticancer effects through apoptosis induction [53]. For the chemical medicines, the compositions are well defined. But for multi-component chemical drugs, especially natural herbs, the chemical composition is usually complex and the main active ingredients are not clear. In fact, natural herbs are a complex system of chemical composition, and the dose–effect relationship is not clear or exists only in a specific concentration range. However, the ingredients of natural herbal medicines are complex, and their effects are the results of the combined effects of various ingredients. Combination of CDDP with phytochemicals can augment the anti-cancer activity of CDDP by triggering apoptosis. In our previous study, RA was the most abundant phytochemicals in *G. hederacea*. But, RA and HWG combination treatment with CDDP on RCC 786-O cells have not been consistent. Therefore it might be other pharmaceutical and nutraceutical components in HWG that would be effective. These results suggest that RA is the major active compound responsible at least in part for the anticancer effect of HWG. We further proceeded to identify the active compound responsible for the cytotoxic activity of the extract by an activity-guided fractionation approach. Our study provides a novel insight on the synergistic use of HWG and RA, natural plant products as chemosensitizers, and cisplatin in the management of 786-O cells. Plant-derived anticancer drugs/natural products are an important resource in the discovery of lead compounds for anti-cancer drug development.

## Conclusion

In conclusion, on the basis of these results we found that CDDP inhibited 786-O cell proliferation and migration. HWG and RA protected against these effects. On the other hand, HWG and RA demonstrate a low cytotoxic effect in HK-2 cells. Cell cycle analysis found that HWG/CDDP and RA/CDDP combined treatment exerted cytotoxicity by inducing G2/M arrest and apoptosis. RA in combined with CDDP significantly inhibited the expression levels of p-FAK (Tyr 925) in RCC 786-O cells in vitro. We propose that the inhibition of RA on RCC 786-O cell invasion and migration may partly occur through the downregulation of FAK phosphorylation. Further studies are needed to test this possibility and extend these findings using in vivo models.

## Data Availability

The datasets used in this study are available from the corresponding author upon reasonable request.

## Abbreviations

CDDP: Cisplatin
DAPI: 4′,6-Diamidino-2-phenylindole
FAK: Focal adhesion kinase
HWG: Hot water extracts of *G. hederacea*
MTT: 3-(4,5-Dimethylthiazol-2-yl)-2,5-diphenylterazolium bromide
PARP: Poly (ADP-ribose) polymerase
PI: Propidium iodide
RA: Rosmarinic acid
RCC: Renal cell carcinoma
TCM: Traditional Chinese medicinal
